# Supplementary figures and images for: Sensitivity Enhancement of Thermometry in Tb3+-Doped KY(CO3)2:Sm3+ by Energy Transfer
Source: Molecules. 2025 Feb 7;30(4):767. doi: 10.3390/molecules30040767 (PMC11857944; doi:10.3390/molecules30040767)

## Supplementary Materials

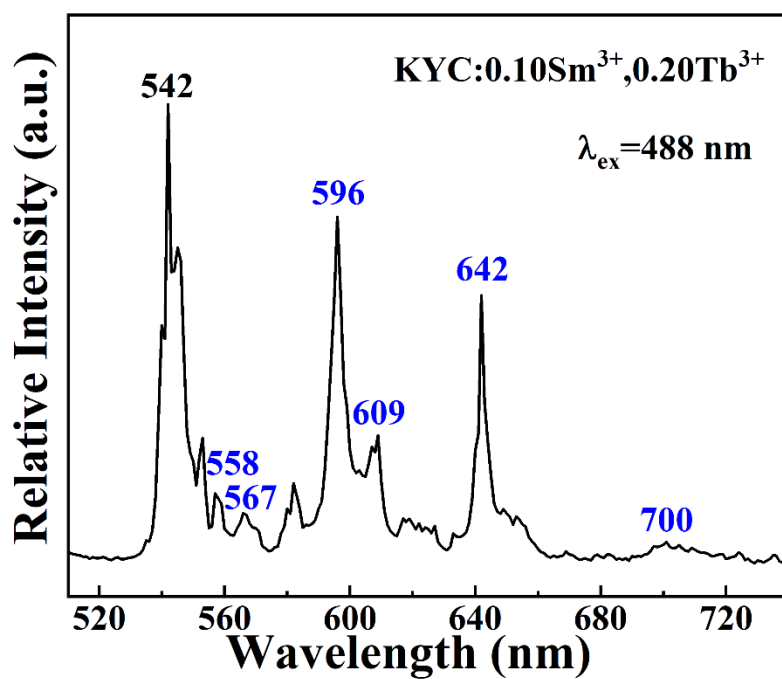

**Figure S1.** Emission spectrum of KYC:0.10Sm<sup>3+</sup>, 0.20Tb<sup>3+</sup> excited by 488 nm.

Supplement: Supplementary file 1 [file molecules-30-00767-s001.zip › molecules-3462994-supplementary.pdf]
